# Synchronous Acquisition and Processing of Electro- and Phono-Cardiogram Signals for Accurate Systolic Times’ Measurement in Heart Disease Diagnosis and Monitoring

**DOI:** 10.3390/s25134220

**Published:** 2025-07-06

**Authors:** Roberto De Fazio, Ilaria Cascella, Şule Esma Yalçınkaya, Massimo De Vittorio, Luigi Patrono, Ramiro Velazquez, Paolo Visconti

**Affiliations:** 1 Department of Innovation Engineering, University of Salento, Road to Monteroni, Building ‘O’, 73100 Lecce, Italy; roberto.defazio@unisalento.it (R.D.F.); ilaria.cascella@unisalento.it (I.C.); suleesma.yalcinkaya@unisalento.it (Ş.E.Y.); massimo.devittorio@unisalento.it (M.D.V.); luigi.patrono@unisalento.it (L.P.); 2 Facultad de Ingeniería, Universidad Panamericana, Aguascalientes 20296, Mexico; rvelazquez@up.edu.mx; 3 Center for Biomolecular Nanotechnologies, Italian Technology Institute IIT, Via Eugenio Barsanti, 14, 73010 Arnesano, Italy; 4 Department of Health Technology, Technical University of Denmark, DK-2800 Lyngby, Denmark

**Keywords:** phonocardiogram, electrocardiogram, systolic times, heart disease monitoring, stethoscope, left ventricle ejection time

## Abstract

Cardiovascular diseases remain one of the leading causes of mortality worldwide, highlighting the importance of effective monitoring and early diagnosis. While electrocardiography (ECG) is the standard technique for evaluating the heart’s electrical activity and detecting rhythm and conduction abnormalities, it alone is insufficient for identifying certain conditions, such as valvular disorders. Phonocardiography (PCG) allows the recording and analysis of heart sounds and improves the diagnostic accuracy when combined with ECG. In this study, ECG and PCG signals were simultaneously acquired from a resting adult subject using a compact system comprising an analog front-end (model AD8232, manufactured by Analog Devices, Wilmington, MA, USA) for ECG acquisition and a digital stethoscope built around a condenser electret microphone (model HM-9250, manufactured by HMYL, Anqing, China). Both the ECG electrodes and the microphone were positioned on the chest to ensure the spatial alignment of the signals. An adaptive segmentation algorithm was developed to segment PCG and ECG signals based on their morphological and temporal features. This algorithm identifies the onset and peaks of S1 and S2 heart sounds in the PCG and the Q, R, and S waves in the ECG, enabling the extraction of the systolic time intervals such as EMAT, PEP, LVET, and LVST parameters proven useful in the diagnosis and monitoring of cardiovascular diseases. Based on the segmented signals, the measured averages (EMAT = 74.35 ms, PEP = 89.00 ms, LVET = 244.39 ms, LVST = 258.60 ms) were consistent with the reference standards, demonstrating the reliability of the developed method. The proposed algorithm was validated on synchronized ECG and PCG signals from multiple subjects in an open-source dataset (BSSLAB Localized ECG Data). The systolic intervals extracted using the proposed method closely matched the literature values, confirming the robustness across different recording conditions; in detail, the mean Q–S1 interval was 40.45 ms (≈45 ms reference value, mean difference: −4.85 ms, LoA: −3.42 ms and −6.09 ms) and the R–S1 interval was 14.09 ms (≈15 ms reference value, mean difference: −1.2 ms, LoA: −0.55 ms and −1.85 ms). In conclusion, the results demonstrate the potential of the joint ECG and PCG analysis to improve the long-term monitoring of cardiovascular diseases.

## 1. Introduction

With cardiovascular diseases ranking as a major cause of mortality worldwide, timely diagnosis and effective monitoring are essential. In recent decades, interest in non-invasive cardiovascular monitoring technologies has grown significantly, driving research toward advanced tools that can improve the diagnosis and clinical management of cardiac disorders. Electrocardiography (ECG) is the gold-standard technique for analyzing the heart’s electrical activity and is widely used to detect rhythm and conduction abnormalities [1,2]. However, ECG alone is not always sufficient to identify all pathological conditions. Disorders related to valvular function, such as heart murmurs, mitral/aortic/tricuspid stenosis, mitral/aortic/ tricuspid regurgitation, etc., cannot be detected solely through electrophysiological analysis; instead, they require an acoustic assessment [3,4].

In this context, phonocardiography (PCG) serves as a valuable complementary tool, enabling the recording and analysis of heart sounds produced during the cardiac cycle. [5,6,7]. These sounds are captured using a phonocardiograph [8]. The mechanical activity of the heart produces four distinct sounds: the first heart sound (S1), second heart sound (S2), third heart sound (S3), and fourth heart sound (S4). S1 coincides with ventricular systole and is characterized by a greater amplitude and longer duration than the other sounds. It is generated by the closure of the mitral and tricuspid valves, lasting between 100 and 200 ms, with a spectral content ranging from 10 to 200 Hz. S1 provides information on the strength of myocardial systole and the functioning of the atrioventricular valves. S2, lasting 100–140 ms, occurs shortly after the beginning of ventricular diastole and coincides with the completion of the T wave in the ECG. It consists of two high-frequency components: one generated by the closure of the aortic valve and the other by the closure of the pulmonary valve; compared to S1, S2 has a higher frequency spectral content. S3 occurs during the rapid filling period of early diastole when blood from the atria fills the ventricles. This period follows the opening of the mitral and tricuspid valves and precedes the slow filling phase (diastasis) and atrial systole. S3 is generated by the vibration of the ventricular walls, which are suddenly stretched due to a backflow of blood caused by the pressure difference between the atrium and ventricle. Finally, S4 occurs in the final phase of diastole, immediately before S1. It is generated by the vibration of the ventricular walls when the atria contract to complete ventricular filling. S4 is generally absent in a healthy heart, but becomes audible due to reduced ventricular compliance [9]. Specifically, increased ventricular wall stiffness leads to a greater atrial contraction force, causing a marked movement of the ventricular walls and, consequently, the generation of S4. In addition to the primary heart sounds, murmurs are acoustic phenomena caused by a turbulent blood flow, which induces vibrations in cardiac structures.

The integration of PCG with ECG analysis has emerged as a promising strategy for enhancing diagnostic capabilities, combining electrical and acoustic assessments for a more comprehensive evaluation of cardiovascular health [10,11]. Recent studies have shown that synchronous ECG and PCG analysis can enhance cardiac pathology detection algorithms, thereby improving the diagnostic accuracy [12].

This study aims to develop an experimental setup for the simultaneous acquisition of ECG and PCG biosignals, accompanied by the development of an adaptive segmentation algorithm to automatically identify the S1, S2, and the onset of S1 sounds in PCG signals and Q-, R-, and S- waves in the ECG signals. These fiducial points were employed to extract the systolic times from synchronous ECG and PCG, including the ElectroMechanical Activation Time (EMAT), the Pre-Ejection Period (PEP), the Left Ventricle Ejection Time (LVET), and the Left Ventricle Systolic Time (LVST), which have been proven useful for the early diagnosis and monitoring of cardiovascular diseases, as discussed in the following Section 4. To ensure the reliability of the proposed systolic time estimation method, the developed algorithm was also validated on an open-source dataset (BSSLAB Localized ECG Data) containing synchronized ECG, PCG, and respiratory signals [13]. The dataset included recordings from multiple subjects, with signals acquired at key auscultation points using both gel and embroidered electrodes, as well as electronic stethoscopes. The algorithm’s outputs—particularly the time intervals between the Q or R peaks and the onset of the S1 heart sound—were compared with reference values reported in the reference paper [14]. This validation step confirmed the consistency and accuracy of the systolic time measurements obtained by the MATLAB-based analysis, reinforcing the system’s potential for clinical and research applications.

The main contributions and novelties of the proposed work are as follows:

- Review the scientific literature regarding wearable systems for discreetly acquiring phonocardiograms and other biosignals for detecting heart diseases.
- Analysis of the main features that can be extracted from the joint analysis of ECG and PCG, highlighting their clinical significance.
- Development of an experimental setup for ECG and PCG’s simultaneous acquisition.
- Development of an adaptive segmentation and feature-extracting algorithm for the joint processing of ECG and PCG based on the segmentation of both biosignals and the extraction of the corresponding systolic times.
- Testing of the developed algorithm on acquired signal and its validation on data provided by an open-source dataset to confirm its reliability.

The remainder of this article is organized as follows: below, the scientific literature on devices and algorithms for detecting and processing heart sounds, as well as systems and algorithms for the joint analysis of ECG and PCG signals, are explored. Section 2 describes the experimental setup used to acquire ECG and PCG simultaneously. Section 3 outlines and explains the MATLAB script used to segment PCG and ECG waves. Additionally, this section analyzes and discusses the extraction of systolic times from the processed PCG and ECG signals, examining the script used. Section 4 discusses the importance of systolic times for heart disease diagnosis, their applications in early cardiovascular disease detection, and the validation of the proposed method for systolic time estimation based on comparison with reference values from an open-source dataset.

### 1.1. Literature Analysis of Advanced Systems to Acquire PCG and ECG

This subsection reports a literature analysis of advanced solutions for PCG and ECG acquisition and processing aimed at early diagnosis and monitoring of heart diseases.

#### 1.1.1. Advanced Devices and Algorithms for Detecting and Processing Heart Sounds

Recent advancements in heart sound acquisition have significantly improved the accuracy and efficiency of cardiac monitoring. Traditional PCG recordings are often susceptible to environmental noise and limited sensitivity, necessitating the development of novel sensors and signal processing techniques. In ref. [15], a bionic MEMS (Micro-Electromechanical Systems) sensor was proposed for heart sound detection at the cardiac auscultation site. The sensor features a cantilever-based design that oscillates in response to acoustic vibrations, causing deformation in a four-branch structure. Each branch incorporates a varistor and the four piezoresistors are configured in a Wheatstone bridge to convert resistance changes into an output voltage, thereby transforming heart sound signals into electrical signals. The sensor demonstrates high sensitivity (−189.5 dB @ 500 Hz), a wide working bandwidth (10–800 Hz), and a high signal-to-noise ratio (29.08 dB), effectively suppressing environmental noise. Finite element simulations were employed to optimize the sensor’s microstructure, while experimental validation confirmed its high-fidelity capability for capturing heart sounds. Such advancements in MEMS technology offer the potential for improving phonocardiographic recordings, thereby enhancing the diagnosis of non-invasive cardiovascular diseases. To further refine heart sound acquisition, researchers have explored alternative sensing techniques. In ref. [16], the authors have developed a microphone array constituted by a thin, low-cost acoustic pressure sensor array for cardiac monitoring. In particular, the array comprises a series of miniaturized electret microphones (ECM-Electret Condenser Microphones), driven by an MCU (Microcontroller Unit) and an FPGA (Field Programmable Gate Array) for the acquisition, analysis, and storage of data from the sensors. The acquired signal undergoes various processing, such as over-sampling and filtering (FIR), which are introduced to reduce noise. The experimental results demonstrate that the proposed system effectively reduces noise, improving the signal-to-noise ratio (SNR) by more than 7 dB. Moreover, the system’s ability to detect S1 and S2 heart sounds was validated by comparing it with a commercial cardiac sound sensor, HKY-06B+. In addition to improving the recording accuracy, making cardiac monitoring accessible to non-medical users is another crucial research aim. A hybrid-type smart stethoscope with a finger-ring design, as presented in ref. [17], enables non-medical users to perform cardiac auscultation independently. The developed device features a foldable ring that can be worn on the finger and positioned on the chest to capture heart sounds as PCG signals (using a digital microphone placed on the lower part of the ring) and photoplethysmography (PPG) signals simultaneously, utilizing an advanced Shannon entropy envelope for pre-processing. An automatic heart sound analysis algorithm determines the time intervals between S1, S2, and PPG peaks. Experimental validation with 20 subjects demonstrated high accuracy in heart sound detection, comparable to professional physiological acquisition systems. This approach significantly broadens access to cardiac monitoring beyond clinical settings. Beyond hardware advancements, advanced signal processing techniques play a critical role in optimizing heart sound analysis. In ref. [18], a method based on the analysis of heart sounds was proposed to assess cardiac fatigue (EICF), exploiting the property of PCG signals to reflect changes in cardiac contractility (inotropy), with a deep learning (DL) network designed to recognize subjects with EICF. A collection of heart sounds was performed in calm subjects and a sports protocol was recorded to obtain intense heart sounds after high-intensity exercise. The heart sounds were used as a dataset to train DL models for EICF diagnosis. A physiological signal collection system established a heart sound database of 1770 samples from 20 athletes aged 19–21. The Discrete Wavelet Transform (DWT) and Logistic Regression Hidden Semi-Markov Model (LR-HSMM) were applied for noise reduction and signal segmentation. A specialized DL network incorporating residual mapping and attention mechanisms was developed, achieving high classification accuracy (98.85%), precision (98.94%), and recall (98.9%). The findings suggest that integrating phonocardiogram-based assessments with advanced machine learning (ML) techniques can enhance cardiac monitoring, complementing ECG-based methods for improved heart disease detection and prevention. Additionally, the study in [19] presents a machine learning-based method for detecting and classifying aortic and mitral stenosis using phonocardiogram (PCG) signals. Features were extracted using DWT, Wavelet Packet Transform (WPT), and Perceptual Wavelet Packet Transform (PWPT) techniques and optimized via Neighborhood Component Analysis. Multiple classifiers were evaluated, including Random Forest, Support Vector Machine (SVM), k-Nearest Neighbors (KNN), and Decision Tree. The Random Forest model achieved the highest accuracy of 99.2%. Validation with data collected from a custom-built device confirmed the model’s effectiveness in real-world scenarios. DL applications in medical audio classification are not limited to phonocardiography alone. The study [20] on the FDC-FS feature-based fusion network presents a DL approach for classifying heart and lung sounds. Traditional methods struggle with complex signal variability, small datasets, and noise. The FDC-FS framework integrates transfer learning from three deep neural network models by transforming audio data into image vectors and fusing the three distinct models into a single, optimized system to address these issues. Using publicly available datasets from the ICHBI (the International Conference on Biomedical and Health Informatics) 2017 challenge and the Heart Challenge data, this study leverages data augmentation techniques, including noise distortion, pitch shifting, and time stretching, to enhance model robustness. Feature extraction focuses on Spectrogram, Mel-frequency Cepstral Coefficients (MFCC), and Chromagram representations, which serve as the input for three convolutional neural network models. Data augmentation enhances the model robustness, achieving 99.1% accuracy for lung sounds and 97% for heart sounds. This study demonstrates that feature fusion and augmentation improve classification and suggests future work on multi-task learning and explainable AI for cardiopulmonary assessments. There are different ways to improve the detection capability by PCG; in fact, in ref. [21], an innovative deep learning-based framework has been proposed that takes into account the individual variability in PCG signals. This study introduces a novel framework to address this issue by clustering patients based on age and PCG time–frequency characteristics. By dividing individuals into subgroups, tailored classification models are developed, improving diagnostic precision. Feature fusion techniques combine MFCCs and PCG signal fragments using convolutional neural networks (CNNs) to extract time–frequency and spatial–temporal features, which are then classified using a Random Forest algorithm. The experimental results show an average classification accuracy of 96.05%, outperforming traditional methods by up to 6.51%. This approach enhances PCG-based CAD (Coronary Artery Disease) diagnosis by reducing individual variability and offers a scalable solution for other biomedical signal applications. Beyond phonocardiography, combining ECG and PCG signals has emerged as a promising strategy for improving CAD detection. In ref. [22], a CNN-based multi-input framework has been developed that integrates features of ECG and PCG signals in the time, frequency, and time–frequency domains for CAD detection. The proposed framework, which utilizes data from 195 subjects, consists of 1D and 2D CNNs. It employs the signals, spectral images, and time–frequency images of ECG and PCG as inputs, demonstrating effectiveness in distinguishing between healthy and CAD subjects. Furthermore, the framework proposed in this study aims to automatically extract multi-domain features from signals with a bi-modal representation (i.e., 1D and 2D). The method achieves a high classification performance with an accuracy of 96.51%, sensitivity of 99.37%, and specificity of 90.08%, outperforming conventional approaches. These results highlight the potential of deep learning-based multi-modal processing for more reliable CAD diagnosis, especially in general medical settings. Similarly, the study in [23] presents a lightweight 1D CNN for classifying PCG signals to screen for valvular heart diseases (VHD). Using self-supervised learning (SSL) for pretraining on unlabeled data, the model achieved high accuracy (up to 99.4%) and strong robustness to noise and signal perturbation. Its compact architecture (only ~20 k parameters) enables real-time inference and smartphone fine-tuning via a progressive web application. Compared to larger models, it significantly reduces the power consumption and processing time, making it practical for mobile health applications. Finally, in [24], the authors propose an automated method for detecting congenital heart diseases (CHDs) in children using phonocardiogram (PCG) signals and a 1D CNN. Data were collected from local clinics and a public dataset, pre-processed, segmented, and augmented via pitch-shifting. The binary classification model distinguishes between normal and abnormal heart sounds. The best-performing model achieved an accuracy of 98.56%, with high sensitivity and precision. The results highlight the potential for real-time CHD screening in pediatric populations using AI-based tools.

A summary of PCG sensors previously discussed is presented in Table 1, which outlines the key characteristics, sensor type, sensitivity, and application areas. This comparison facilitates a deeper understanding of the available sensor technologies and their suitability for specific phonocardiography use cases.

Additionally, Table 2 summarizes the previously discussed algorithms, highlighting key aspects, including the main objectives, such as the approach or model, output classes, input features, and accuracy of each algorithm. This comparative analysis offers insights into the strengths and limitations of various methodologies, thereby supporting the selection of suitable methods for future research and system enhancements.

#### 1.1.2. Systems and Algorithms for Joint Analysis of ECG and PCG Signals

Recent advancements in cardiovascular monitoring have focused on integrating ECG and PCG for improved disease detection and pre-screening accuracy. A wearable multi-channel ECG-PCG device developed by researchers in collaboration with Ticking Heart [25] utilizes an iterative Wiener filter-based noise cancellation algorithm to enhance the integrity of the heart sound signal, particularly in the 200–300 Hz range. This approach demonstrates superior noise suppression compared to existing techniques. Furthermore, a CNN-based classifier has been implemented to jointly analyze ECG and PCG signals, significantly improving the cardiovascular disease (CVD) detection accuracy over PCG-only methods. These findings highlight the potential of DL algorithms and advanced signal processing techniques in optimizing joint ECG-PCG analysis to monitor heart disease.

Another approach involves a multi-sensor array designed for the home monitoring of patients with chronic heart failure (CHF) [26]. This flexible array, developed for inexperienced users, integrates 48 microphones for high-resolution PCG recording, ECG electrodes, and a magneto-inertial measurement unit to allow simultaneous ECG-PCG acquisition. Experimental validation with 42 volunteers demonstrated the feasibility of obtaining high-quality signals regardless of anatomical variations, overcoming challenges related to sensor placement. The system enables the extraction of cardiac time intervals (CTIs), providing insights into intracardiac pressures and changes in cardiac function. Unlike conventional CHF monitoring methods, which primarily detect decompensation at later stages, this approach offers a proactive solution for early detection in a home care setting. Despite limitations such as reliance on a nonstandard single-lead ECG and a relatively small study sample, the findings suggest significant potential for telemonitoring applications. Future research will focus on validating the system in CHF patients and broader cardiovascular conditions to establish its clinical relevance further. Similarly, the study in [27] developed a novel risk-scoring system for early heart failure screening using data from 813 participants. It combined ECG, PCG, and radial artery hemodynamic features, including EMAT, S4, A2-P2, RV5 + SV1, and SBP. The score showed a strong diagnostic performance with AUCs of 0.802 (training set) and 0.762 (testing set). This system may help primary care providers detect heart failure early and guide timely intervention.

Another innovative approach for detecting heart disease is the Gaussian Kaiming Variance-based Deep Learning Neural Network (GKVDLNN), which combines ECG and PCG signals using advanced signal processing techniques to improve the diagnostic accuracy [28]. The approach overcomes the limitations of traditional models by utilizing large datasets and an improved empirical mode decomposition (IEMD) technique for feature extraction. By selecting and concatenating the most relevant features, the system enhances the classification performance, achieving an impressive accuracy of 96.103%. Compared to existing heart disease detection frameworks, GKVDLNN demonstrates superior reliability, reducing false negatives and ensuring a consistent performance. Also, CNNs demonstrated their suitability in processing ECG and PCG signals. Indeed, in [29], the authors propose a hybrid CNN-LSTM model for detecting cardiac abnormalities from ECG and PCG signals. Using the PhysioNet 2016 and 2017 datasets, signals were pre-processed via Empirical Mode Decomposition (EMD) and converted into scalograms for feature extraction. The CNN captured spatial features, while the LSTM modeled temporal dependencies. The model achieved 86% overall accuracy with high precision and recall in classifying normal and abnormal heart signals. The results support its potential for the early, automated diagnosis of cardiovascular diseases. However, while these methods effectively classify heart disease using ECG and PCG signals, they do not incorporate additional patient data such as medical history or vital signs, which could further refine their diagnostic capabilities. Future research should focus on integrating these complementary data sources to develop a more comprehensive and precise heart disease detection system. Another advancement in multi-modal monitoring is the development of a wearable stethoscope for simultaneous ECG and respiratory signal estimation proposed in the ref. [30]. This system integrates a 55 mm single-lead ECG and a stethoscope to estimate Einthoven ECG Lead I and II, the pre-ejection period (PEP), the left ventricular ejection time (LVET), and respiratory parameters. This study demonstrated high correlation values exceeding 0.97 for ECG estimations, while respiratory rate estimations showed a mean absolute error of less than 1.2 bpm. Despite some limitations related to subject positioning and intersubject variability, the device offers a promising, non-invasive approach for the long-term monitoring of cardiovascular and respiratory conditions.

Future research is needed to develop subject-specific regression models and assess the system’s long-term applicability through polysomnography evaluations. This research highlights the potential of wearable, multi-modal acquisition systems for enhancing the early detection and continuous monitoring of cardiovascular and respiratory conditions. Table 3 provides a comparison of the analyzed systems used for acquiring and processing PCG and ECG signals, detailing the key components, including the number of acquisition channels, types of PCG sensors, application areas, additional acquired signals, inference algorithm used, and measurement or classification types. This comparison highlights each system’s unique features and methodologies, allowing for a deeper understanding of their advantages and challenges. This analysis is crucial for defining the architecture and functionalities of next-generation diagnostic systems for heart disease.

## 2. Materials and Methods 

This section outlines the hardware components, electronic configuration, and experimental setup designed to acquire PCG and ECG signals simultaneously. A custom-built system was assembled using commercially available sensors and development boards. The PCG signal was acquired using an electronic stethoscope (model HM-9250, manufactured by HMYL, Anqing, China), while the ECG signal was recorded using the AD8232 ECG sensor module (manufactured by Analog Devices, Wilmington, MA, USA). Both signals were synchronized and digitized using an Arduino Nano BLE development board (manufactured by Arduino Co., Monza, Italy) and transmitted via USB to a host computer for visualization and storage. The subsequent segmentation and analysis of PCG and ECG signals were performed to extract cardiac features and calculate systolic times. Data were recorded using a stethoscope connected to a PC sound card and simultaneously acquired via an Arduino Nano BLE (manufactured by Arduino Co., Monza, Italy). A MATLAB script processed the signals, identifying the onset and peaks of S1 and S2 heart sounds in the PCG and the Q, R, and S waves in the ECG. The accuracy of the segmentation was validated through visual inspection, confirming the reliability of the acquisition system.

### 2.1. Description of the Used Materials and Devices

**Figure 1:**
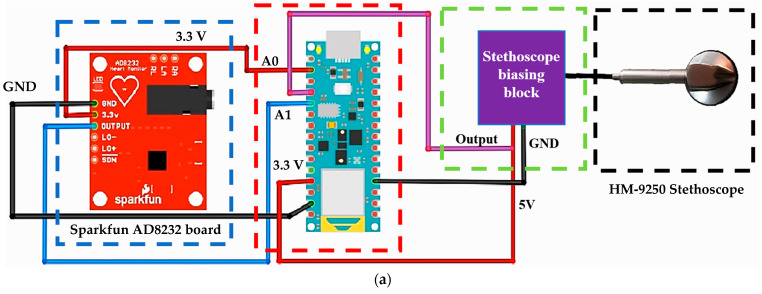

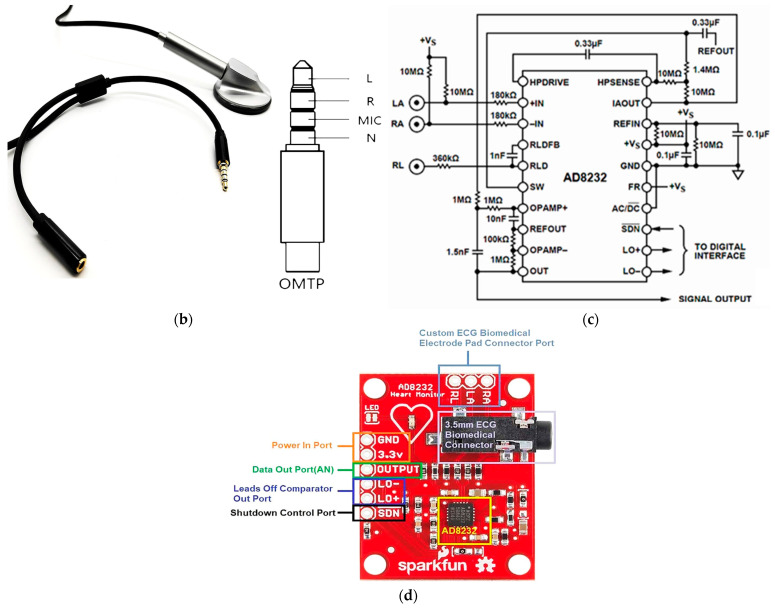
Setup for ECG and PCG acquisition with the used components highlighted: SparkFun AD8232 board (blue box), Arduino Nano BLE (red box), biasing block for electronic stethoscope (green box), and the HMYL HM-9250 stethoscope (black box) (**a**). HMYL HM-9250 stethoscope and pinout of the TRRS connector with OMTP standard integrated into the stethoscope (**b**). Typical connection diagram of the AD8232 integrated circuit for ECG measurement (**c**). SparkFun AD8232 ECG module (**d**).

The electronic stethoscope HMYL model HM-9250, used to record the PCG signal, is primarily designed to detect sounds that can be heard on the body’s surface, such as dry and wet lung rales (Figure 1b), but it is also suitable for picking up heart, breathing, bowel, and other sound signals. The HM-9250 stethoscope includes an electret microphone and a biasing circuit powered by a bias voltage, as reported in Table 4. The voltage was applied via the 3.5 mm^2^ TRRS connector following the OMTP (Open Mobile Terminal Platform) standard, using the dedicated microphone pin from which the audio output was extracted (Figure 1c).

The AD8232 chip, developed by Analog Devices, was integrated into the SparkFun AD8232 ECG board (manufactured by SparkFun Electronics, Niwot, CO, USA) to acquire the ECG signal. The AD8232 features a Common Mode Rejection Ratio (CMRR) of 80 dB in the 0–60 Hz band and supports both two-electrode and three-electrode configurations. The device operates within a supply voltage range of 2–3.5 V. Figure 1c provides a top view of the board, describing its connections and integrated components. The board includes a 3.5 mm jack connector, a status LED, and the AD8232 IC. The evaluation board’s pin functions, necessary for interfacing with a microcontroller unit, include an analog pin for the sensor output and three digital pins for monitoring the state of the control pins provided by the board.

The Arduino Nano BLE development board manages the devices mentioned above based on the nRF52840 System on Chip (SoC) produced by Nordic Semiconductor. Designed for IoT applications and embedded systems, this platform integrates a Bluetooth Low-Energy (BLE) transceiver, enabling communication with BLE-compatible devices such as smartphones, tablets, and other microcontrollers. The nRF52840 SoC (manufactured by Nordic Semiconductor, Trondheim, Norway), built on a 32-bit ARM Cortex-M4 architecture (manufactured by STMicroelectronics, Geneva, Switzerland), supports a clock frequency of up to 64 MHz and includes 256 KB of flash memory for code storage and 32 KB of RAM for temporary data management. The Arduino Nano BLE board measures 45 mm × 18 mm, making it ideal for space-constrained applications. Additionally, it features a USB interface for programming and serial communication with a host computer. Among its additional features, the board integrates a 9-axis inertial sensor and an integrated microphone, which can be used for motion detection and voice recognition. The board can be supplied via a USB connection or through pins for external power sources.

### 2.2. Description of the Experimental Setup for Simultaneous Acquisition of ECG and PCG

The setup for the simultaneous acquisition of PCG and ECG signals consists of an electronic stethoscope (model HM-9250, manufactured by HMYL Ltd., Figure 1b) for PCG signal auscultation. The stethoscope relies on an electret condenser microphone with a frequency response of 20 Hz to 20 kHz, a sensitivity of −52 ± 2 dB, and an output signal range of 0–200 mV. It operates with a 2.6–3 V DC bias voltage, providing approximately 4× signal magnification. The microphone generates an analog output and maintains a white noise level lower than –45 dB (relative to mobile phone audio standards). The signal is sampled at 44.1 kHz, following either the National Standard or American Standard interface conventions through a 3.5 mm TRRS (“tip-ring-ring-sleeve”) connector (OMTP standard—Open Mobile Terminal Platform). In the experimental setup, the stethoscope is powered via a 5 V supply from the Arduino Nano BLE board and connected to the microphone pin through a 12 kΩ pull-up resistor to provide proper biasing. The resulting audio signal is acquired from the same pin using analog input A0 (Figure 1a).

The SparkFun AD8232 ECG electronic board, an evaluation board of the AD8232 IC (manufactured by Analog Device, Wilmington, MA, USA), was used in the setup to detect the ECG signal. Figure 1d shows the top view of the SparkFun AD8232 board with a description of the connections and components on the board. The electronic board has a 3.5 mm jack connector, a status LED, and the AD8232 integrated circuit. The AD8232 integrated front-end features a Common Mode Rejection Ratio (CMRR) of 80 dB (in the 0 to 60 Hz band), can be used in a two- or three-electrode configuration, and operates with a power supply in the range of 2 to 3.5 V. Figure 1c shows a typical connection scheme for the AD8232 IC used in the ECG acquisitions. In detail, the SparkFun AD8232 board, powered by 3.3 V DC from the Arduino board, conditions the ECG signal, which is acquired through an analog input (A1) (Figure 1a). The Arduino Nano BLE board is connected to a PC via USB, providing power and a virtual serial communication interface. Through this USB connection, the board transmits the acquired and filtered PCG and ECG signals in real time, allowing them to be visualized and stored using serial monitoring tools such as SerialPlot or custom data acquisition scripts.

**Figure 2:**
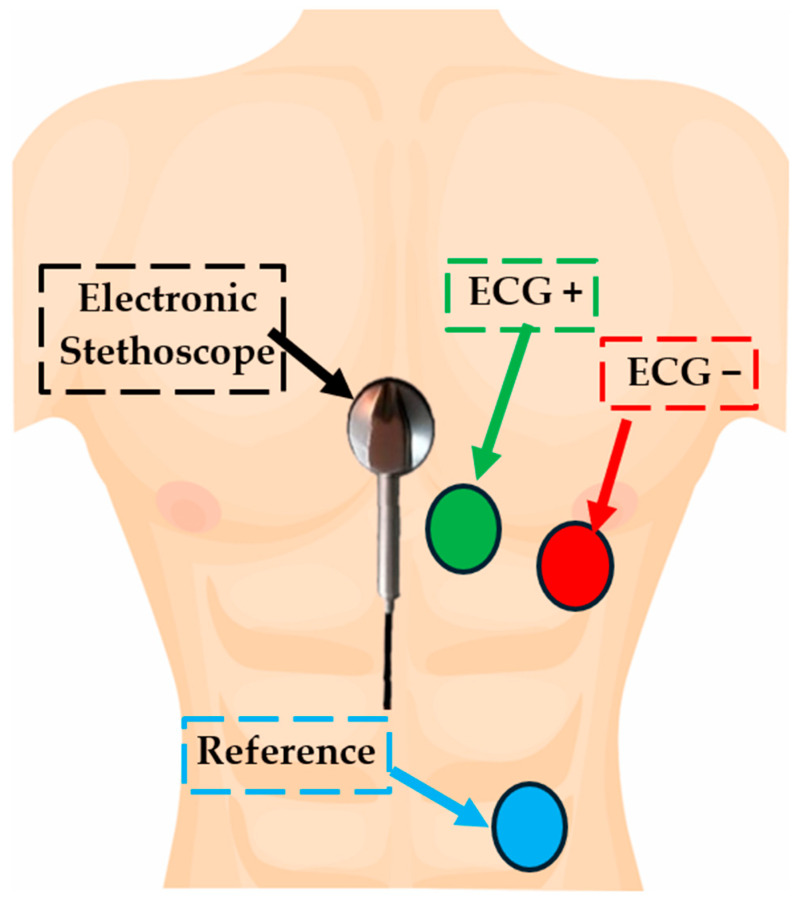
Positioning of the electronic stethoscope for PCG signal acquisition and the electrodes for three-lead ECG recording (ECG+, ECG−, Reference).

The acquisition and analysis of the signals were performed on a single subject in a resting position, seated on a chair. The electrodes for ECG detection were applied to the subject as follows: on the right side of the chest (ECG+), on the left side of the chest (ECG−), and on the lower left abdomen (Reference), as shown in Figure 2. The stethoscope for PCG acquisition was positioned at the center of the chest and secured with adhesive tape. Then, the signals were acquired and stored in a .csv file using the SerialPlot serial plotter.

The code implemented on the Arduino Nano BLE board begins by including the Filters.h library, which allows the application of digital filters to real-time signals (https://github.com/JonHub/Filters, accessed on 20 January 2025). Two second-order low-pass digital filters are applied: a 100 Hz cut-off filter for the PCG signal and a 15 Hz cut-off filter for the ECG signal. After initializing the UART interface with a baud rate of 115,200 baud, the ADC resolution is set to 12 bits (lines 5–6).

In the main loop, the first operation consists of acquiring the ECG and PCG signals. To obtain a synchronized estimate of the PCG signal with the ECG signal, the PCG signal is acquired before and after the ECG sample, and the average of the two PCG samples is used as the estimated PCG sample synchronized to the ECG (line 13). Subsequently, the PCG and ECG signal samples are processed through their respective low-pass filters to reduce noise. Finally, the filtered signal samples are sent over the microcontroller’s UART interface and the corresponding timestamp (lines 18–22).
1 #include **<**Filters.h**>** 2 FilterTwoPole filterTwoLowpass_pcg**(**100**,**1**,**0**);** // set the LP filter for PCG 3 FilterTwoPole filterTwoLowpass_ecg**(**15**,**0.707**,**0**);**// set the LP filter for ECG 4  **void** setup**() {** 5  Serial.begin**(**115200**);** 6  analogReadResolution**(**12**);**
7  analogReference**(**AR_VDD**);** 8 **}** 9 **void** loop**() {** 10 **int** raw_pcg= analogRead**(**A0**);** 11 **int** ecg = analogRead**(**A1**);** 12 **int** raw1_pcg= analogRead**(**A0**);** 13 **float** pcg = 0.5*(raw_pcg + raw1_pcg**);**//estimates the synchronous sample of PCG with respect to the ECG 14 filterTwoLowpass_pcg.input**(**pcg**);** //2nd-order LP filter for PCG 15 **float** filtered2 = filterTwoLowpass_pcg.output**();** // provide the output of LP filter 16 filterTwoLowpass_ecg.input**(**ecg**);** // 2nd-order LP filter for ECG 17 **float** ecg_filtered = filterTwoLowpass_ecg.output**();** //provide the output of the LP filter 18 Serial.print**(**millis**());** 19 Serial.print**(**“,”**);** 20 Serial.print**(**filtered2**);** 21 Serial.print**(**“,”**);** 22 Serial.println**(**ecg_filtered**);** 23 delay**(**1**);** 24 **}** 

To further enhance the timing precision and real-time performance of the synchronous ECG-PCG acquisition system, future work will implement hardware timer interrupts on the nRF52840 microcontroller, replacing the current delay-based sampling approach. This modification will ensure consistent sampling intervals, minimize jitter, and improve the system’s ability to handle concurrent tasks such as filtering and wireless data transmission, thereby increasing the reliability of systolic time measurements for long-term cardiovascular monitoring.

### 2.3. Description of the MATLAB Script for Segmenting PCG and ECG

**Figure 3:**
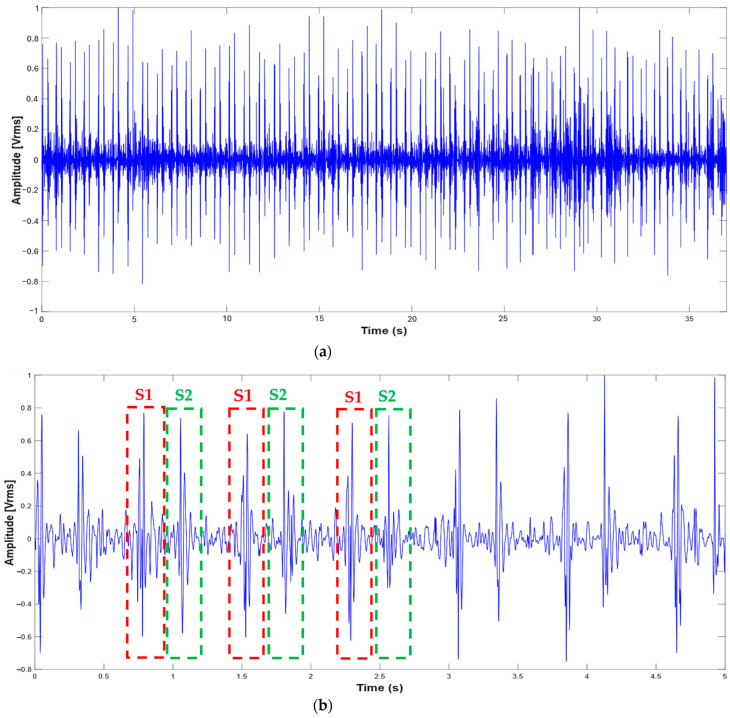
PCG signals provided by the stethoscope and acquired via the PC sound card: 37 s (**a**) and 5 s traces (**b**).

**Figure 4:**
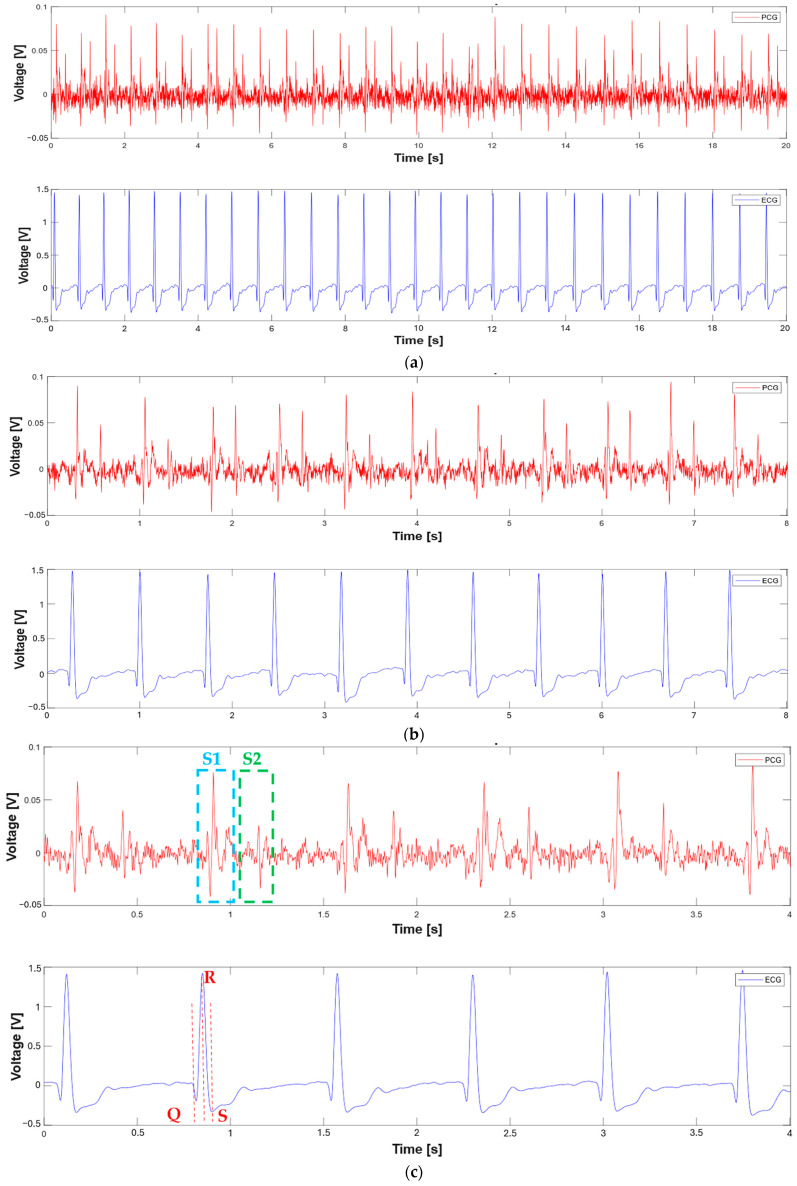
PCG and ECG signals acquired simultaneously at varying durations: 20 s (**a**), 8 s (**b**), and 4 s (**c**).

Before performing any processing or analysis, it was crucial to verify the proper functioning of the stethoscope. To achieve this, the PCG signal was acquired by directly connecting the stethoscope to the PC’s sound card. The signals were recorded using Audacity audio acquisition software (version 3.7.3), stored in .wav format, and visualized using a custom MATLAB script. Figure 3 displays two recorded traces (37 and 5 s in duration), demonstrating clear and distinguishable S1 and S2 heart sounds within each cardiac cycle (Figure 4). Following this, the experimental setup described in Section 2 was tested by simultaneously acquiring PCG and ECG signals. The data, collected via the Arduino Nano BLE’s serial interface, were logged in a .csv file using SerialPlot software (version 0.13) and later processed and plotted in MATLAB (version 24.1). As the subsequent figures show, both signals retained their defining characteristics: the PCG exhibited well-defined S1 and S2 sounds, while the ECG displayed clearly identifiable QRS complexes. This observation confirms the integrity of the acquisition system for subsequent analysis.

The analysis begins by loading the recorded data from the .csv file, which contains timestamped PCG and ECG signals acquired via the Arduino board’s serial interface. The signals are processed over a 10 s time window with no overlap; thus, the signal slice and the time axis are extracted from the file containing PCG and ECG signals. Then, the DC component is removed from both signals by subtracting their mean values over the observation window and detrending them (lines 20 and 27). A 2-Hz high-pass IIR (Infinite Impulse Response) filter is implemented (line 21) and applied to the ECG signal to remove baseline wander caused by body movements during acquisition (line 26).
**for** s **=** 1**:**1**:**window_number 19  t **=** data**.**b**(**slice_*(s**-**1**) +** 1**:**slice_*s**,**1**);** %time loading 20   ecg **=** detrend**(**data**.**b**(**slice_*(s**-**1**) +** 1**:**slice_*s**,**8**));** %ecg loading 21  hpFilt **=** designfilt**(**‘highpassiir’**,** ... 22 ‘FilterOrder’**,** 4**,** ... 23 ‘HalfPowerFrequency’**,** 2**,** ... 24 ‘SampleRate’**,** fs**);** 25  % Apply the filter 26  ecg **=** filtfilt**(**hpFilt**,** ecg**);**% ecg filtering 27  pcg **=** detrend**(**data**.**b**(**slice_*(s**-**1**) +** 1**:**slice_*s**,**3**)-**     Mean**(**data**.**b**(**slice_*(s**-**1**) +** 1**:**slice_*s**,**3**)))** 

**Figure 5:**
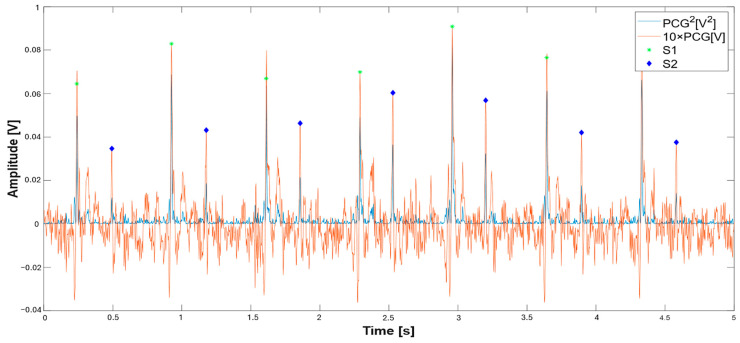
PCG signal segmentation based on squared PCG signal: squared PCG signal (blue trace), PCG signal (orange trace). S1 sounds (green asterisk) and S2 sounds (blue diamonds).

For PCG processing, the algorithm segments the signal to identify the onset and peaks of S1 and S2 heart sounds, leveraging morphological and temporal features of the PCG signal. In detail, the squared PCG signal (blue trace, Figure 5) enhances S1/S2 detection by emphasizing the signal’s peaks, with each cardiac cycle exhibiting two distinct peaks corresponding to the S1 and S2 heart sounds; notably, the amplitude of the S2 sounds is significantly lower than that of S1. For S1 sound detection, a dynamic threshold equal to $A_{S1,\;th}=0.1*max(PCG^{2})$ is applied to the PCG signal, identifying the times the signal is higher than that threshold. Subsequently, a logic signal is generated where the condition is satisfied, and the centers of those time intervals are determined (lines 63); also, an amplitude threshold equal to $B_{S1,\;th}=0.5*\bar{\left(PCG(S1\right)}$, where PCG(S1) is the value calculated to the determined peaks, is applied. Finally, a time threshold is used to select peaks more than 425 ms apart (line 67). The dual condition on amplitude and time allows reliable detection of the S1 peaks.
48 A_S1_th **=** 0.1*max**(**pcg**.^**2**);**% parametric threshold on S1 sound 49 peak_S1 **=** find**(**pcg**.^**2 **>=** A_S1_th**);** %find S1 peak 50 S1_sign **=** generate_signal**(**peak_S1**,**length**(**pcg**));**%generate logic signal 51  figure**(**2**);** 52  fig_ **=** gcf**;** 53  fig_**.**WindowState **=** ‘maximized’**;** 54  subplot**(**2**,**1**,**1**)** 55  plot**(**t**,(**pcg**.^**2**));**hold on 56  xlabel**(**‘Time [s]’**,** ‘fontweight’**,** ‘bold’**,** ‘fontsize’**,**16**);** 57  ylabel**(**‘Amplitude [V^2]’**,** ‘fontweight’**,** ‘bold’**,** ‘fontsize’**,**16**);** 58  legend**(**‘PCG^2[V^2]’**,**’S1’**,**’S2’**);** 59  subplot**(**2**,**1**,**2**);** 60  plot**(**t**,** pcg**);**hold on 61  %xlim([0 11]) 62  S1_time **=** find_center_points**(**S1_sign**);**% find center of logic signal 63  B_S1_th **=** 0.5*mean**(**pcg**(**S1_time**));**% amplitude threshold on S1 detection 64  S1_time **=** S1_time**(**pcg**(**round**(**S1_time**)) >=** B_S1_th**);** % amplitude filtering on S1 65  differences **=** diff**(**S1_time**);** 66  threshold **=** 0.425*fs**;** 67  keep_index **= [**true**,**abs**(**differences**) >=** threshold**];** %time filtering on S1 sounds 68  S1_time **=** S1_time**(**keep_index**);** 

**Figure 6:**
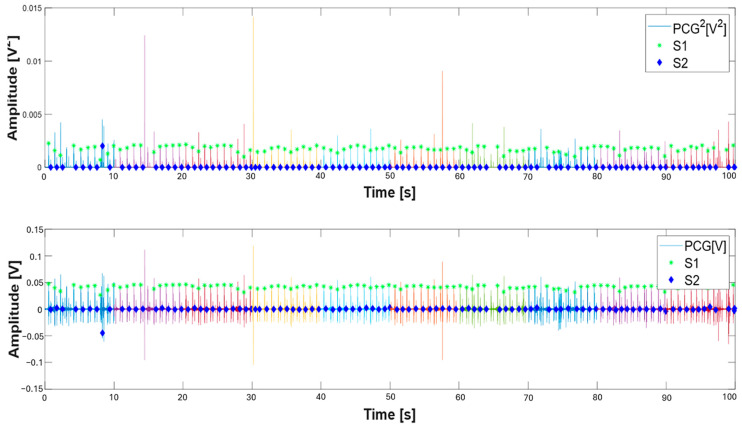
PCG segmentation based on the processing of the squared signal: Squared-PCG and PCG signals with the identified S1 (green asterisk) and S2 locations (blue diamonds).

Starting from the detected locations of the S1 sounds, the corresponding S2 sounds are searched within the squared PCG signal (line 85). Time constraints are applied, requiring that the interval between S1 and S2 falls within 125 ms to 500 ms (line 87), values considered safe margins for detecting S2 sounds. Later, the resulting S1 and S2 sound locations are assessed; the approach focuses on amplitude consistency as the primary validation criterion; S1 detections are accepted only if their amplitude falls within 40% of either a running average of previous S1 amplitudes or a default threshold imposed according to PCG amplitude. A high tolerance of 40% is introduced to account for the natural oscillations in real PCG signals. This approach makes sense physiologically since heart sounds from the same source (the mitral and tricuspid valve closures) should have consistent amplitudes under stable conditions. Figure 5 presents the segmentation of the PCG signal, showing both the squared PCG signal (orange trace) and the PCG signal multiplied by a factor of 10 to make the latter comparable to the quadratic signal; the identified peaks of S1 and S2 sounds are highlighted (green asterisks and blue diamonds, respectively). Figure 6 depicts the trends of squared-PCG and PCG signals with a time duration of 100 s, highlighting the identified S1 and S2 sound locations.
% Detection of S2 sounds 70 %Determines ranges around the S1 sounds 71 **for** j **=** 1**:**length**(**S1_time**)** % Prevent index overflow at j + 1 72  % Ensure range indices are integers 73   **if** j **<** length**(**S1_time**)** 74    start_idx **=** round**(**S1_time**(**j**));** 75    end_idx **=** round**(**S1_time**(**j **+** 1**));** 76   **elseif** j**==**length**(**S1_time**)** 77    start_idx **=** round**(**S1_time**(**j**));** 78    end_idx **=** length**(**t**);** 79  **end**
80   **if** start_idx **>=** end_idx **||** end_idx **>** length**(**pcg**)** 81    S2_time**(**j**) =** NaN**;** % Or handle appropriately 82   **continue;** 83  **end** 84   range **=** start_idx**:**end_idx**;** 85  **[~,**peak_S2**] =** findpeaks**(**pcg**(**range**).^**2**);**% find S2 sounds on squared PCG 86   differences1 **=** peak_S2**;** % Relative to start of range 87   indices_logical**= (**differences1 **<** 0.5*fs**) & (**differences1 **>** 0.125*fs**);** %time filtering 88   peak_S2_index **=** find**(**indices_logical**);** 89  **if ~**isempty**(**peak_S2_index**)** 90    peak_S2 **=** peak_S2**(**peak_S2_index**);** 91    S2_time**(**j**) =** mean**(**start_idx **+** peak_S2**);** % Convert back to full signal index 92    **else** 93      S2_time**(**j**) =** NaN**;** % No valid peaks found 94    **end** 95  **end** 

**Figure 7:**
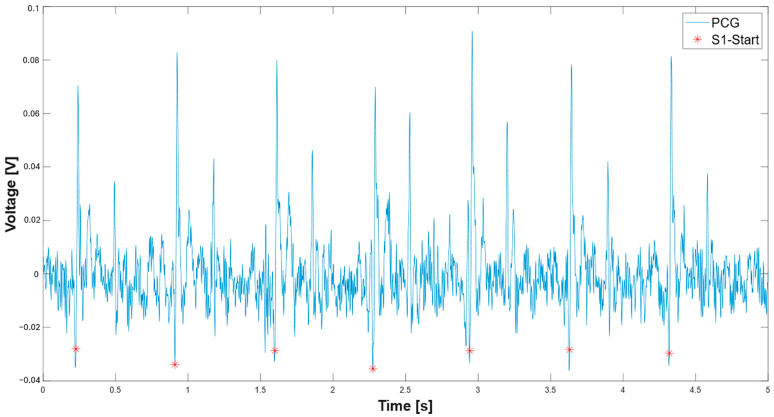
Detection of the start of the S1 signal of the PCG signal (red asterisk).

Subsequently, the script processes the PCG signal to detect the S1 onset. First, a low-pass IIR filter with a 40 Hz cut-off frequency is applied to reduce noise and extraneous components superimposed on the signal and to make the initial transition of the S1 sound clearer. The script processes the inverted PCG signal (line 205) using MATLAB’s findpeaks function to detect peaks. It applies a minimum amplitude threshold of 0.2×max(−PCG) and enforces a minimum peak-to-peak interval of 200 ms (line 207). After detecting potential S1 peaks in the inverted signal, the script performs a backward search to locate the exact onset of each S1 sound. For each detected peak, it traces backward through the signal until it finds where the signal stops decreasing (i.e., where the slope changes). This approach gives a more accurate onset time than just the peak location. Then, the script validates these detected onset times against the known S1 locations (S1_time_). It uses a tolerance window of 50 ms to match onset times with known S1 locations, keeping only those onsets sufficiently close to a known S1. This two-stage approach—detecting potential onsets and validating them against known S1 locations—helps reduce false positives. The segmentation results of the PCG signal (blue trace) and the identified S1 onset markers (red asterisks) are illustrated in Figure 7.

**Figure 8:**
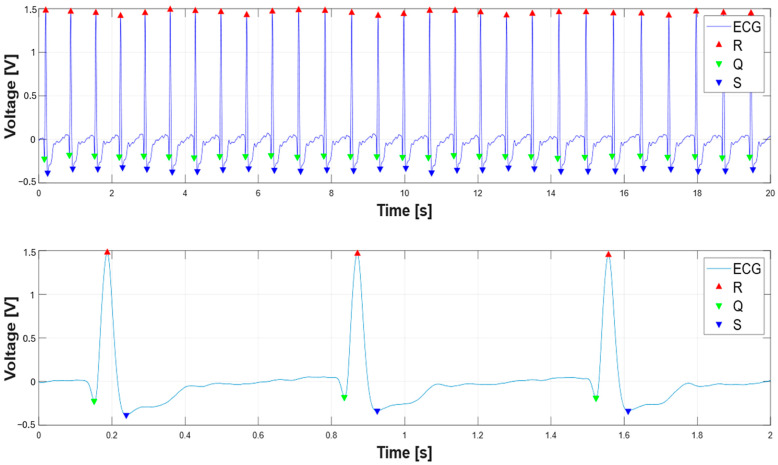
Segmented ECG signal to detect Q (green triangle), R (red triangle), and S wave (blue triangle) peaks.

Afterward, the script performs ECG segmentation by detecting key features of the QRS complex, specifically the R-peaks, Q-peaks, and S-peaks. The approach combines straightforward peak detection with clever adaptive techniques to handle real-world ECG variability. The script starts with the R-peak detection, using a threshold set at half the maximum ECG amplitude ($A_{R-peak,th}=0.5*max(ECG)$). This approach works reasonably well because R-peaks are typically the most prominent feature in an ECG signal. The “findpeaks” function is applied alongside a time vector, ensuring the preservation of temporal context throughout the analysis. Afterward, the script employs a methodical approach to Q and S peak detection by establishing fixed-duration search windows relative to each identified R-peak. Specifically, it examines a window extending 250 ms preceding the R-peak for Q-wave identification and 250 ms following the R-peak for S-wave detection. In particular, an adaptive threshold is employed for Q and S wave detection, equal to $A_{Q,S-peak,th}=0.2*max(-ECG)$, along with a minimum distance for S-wave detection proportional to the previously introduced time range span around the R-peak. Figure 8 shows the segmentation results of an ECG signal with a 10 s time duration, with highlighted locations of the identified Q, R, and S peaks.

**Figure 9:**
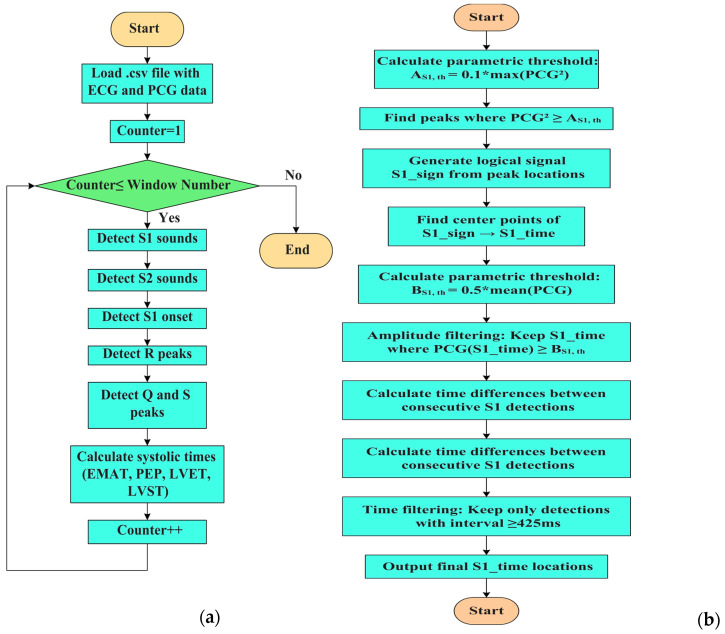

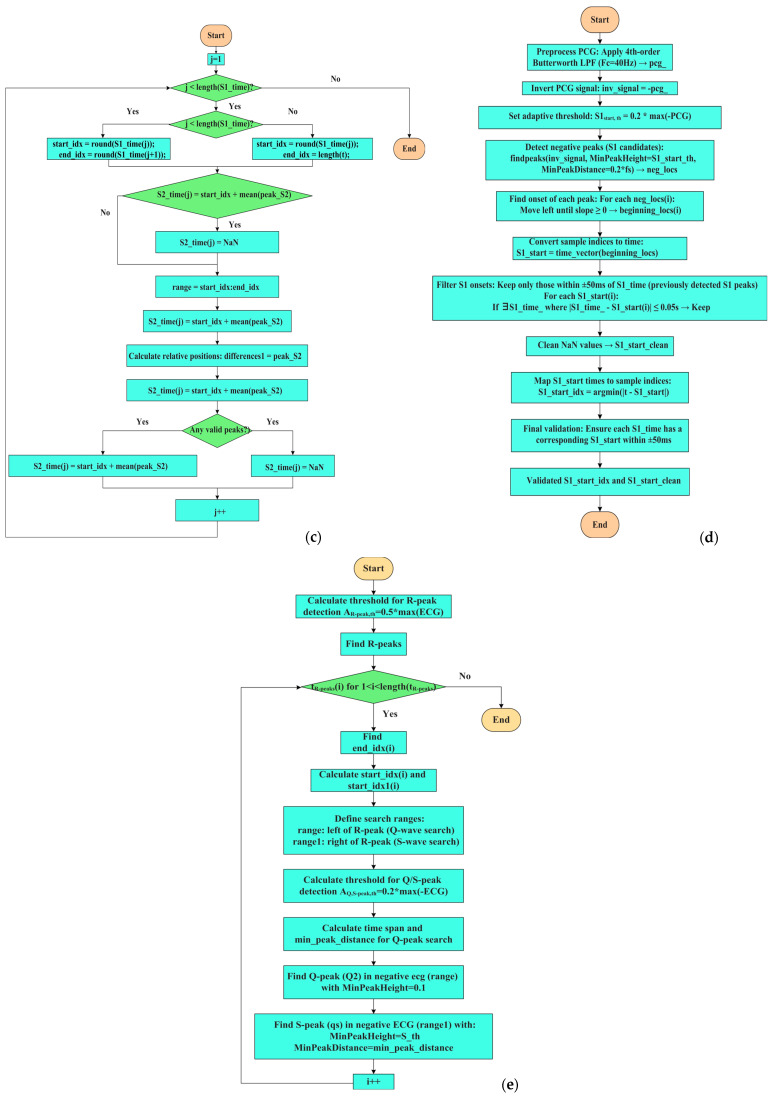
Flowcharts of the MATLAB script developed for segmenting ECG and PCG signals and calculating systolic time intervals: the overall high-level structure of the segmentation algorithm of PCG and ECG signals (**a**); detection of S1 sounds (**b**); detection of S2 sounds (**c**); and identification of the S1 onset of PCG signal (**d**). Identification of Q and S peaks of the ECG signal (**e**).

In summary, the high-level flowchart of the developed MATLAB script is reported in Figure 9a, where the main implemented steps are synthesized. Additionally, Figure 9b–d presents the flowchart of the developed script for identifying S1 and S2 sounds and S1 sound onsets, which follow the operating modalities described above.
203  min_peak_distance_sec **=** 0.2**;** 204  min_peak_distance_samples **=** round**(**min_peak_distance_sec * fs**);** 205  inv_signal **= -**pcg**;**% inversion of the signal 206  S1_start_th **=** 0.2*max**(-**pcg**);**% adaptive threshold on S1 onset 207  **[**neg_peaks**,**neg_locs**] =** findpeaks**(**inv_signal**,**’MinPeakHeight’**,** S1_start_th**,**’MinPeakDistance’**,**min_peak_distance_samples**)** %find peaks on inverted signal**;** 208    % Find beginning of each negative peak 209    beginning_locs **=** zeros**(**size**(**neg_locs**));**
210    **for** i **=** 1**:**length**(**neg_locs**)** 211      idx **=** neg_locs**(**i**);** 212  %Go backward until slope is no longer negative (signal starts falling) 213    **while** idx **>** 1 **&&** pcg**(**idx**) <** pcg**(**idx **-** 1**)** 214      idx **=** idx **-** 1**;** 215    **end** 216      beginning_locs**(**i**) =** idx**;** 217    **end** 218    time_vector **= (**0**:**length**(**pcg**)-**1**) /** fs**;** 219    peak_times **=** time_vector**(**neg_locs**);** 220    S1_start **=** time_vector**(**beginning_locs**) +** t**(**1**);** 


%Find ECG peaks

270  R_peak_th **=** 0.5*max**(**ecg**);**

271  **[**R2**,**TR2**] =** findpeaks**(** ecg**,** t**,** ‘MinPeakHeight’**,** R_peak_th**);**

272  **for** i **=** 1**:**length**(**TR2**)**% starting from R-peak search for Q and S peaks

273    end_idx **=** find**(**t **==** TR2**(**i**));**

274    **if** isempty**(**end_idx**)**

275      **continue;** % Skip if t doesn’t contain exact TR2(i)

276    **end**


277    start_idx **=** end_idx **-** 0.250*fs**;**

278    start_idx **=** max**(**start_idx**,** 1**);** % Ensure within bounds

279    start_idx1 **=** end_idx **+** 0.250*fs**;**

280    start_idx1 **=** min**(**start_idx1**,** length**(**t**));** % Ensure within bounds

283    range **=** start_idx**:**end_idx**;** %range to left

284    range1 **=** end_idx**:**start_idx1**;**%range to right

286    **if** isempty**(**range**) ||** isempty**(**range1**)**

287       **continue;** % Prevents findpeaks on empty vector

288    **end**

289    S_th **=** 0.2*max**(-**ecg**);** %threshold on S-peaks

290    range_time_span **=** t**(**range1**(**end**)) -** t**(**range1**(**1**));** % time threshold for Q-peak search

291    min_peak_distance **=** min**(**0.05**,**0.9*range_time_span**);** % for safety

292    **[**Q2**(**i**),**TQ2**(**i**)] =** findpeaks**(-**ecg**(**range**),**t**(**range**),**’MinPeakHeight’**,** 0.1**);** %find Q-peak

293    **[**qs**,** ts**] =** findpeaks**(-**ecg**(**range1**),** t**(**range1**),** ...

294      ‘MinPeakHeight’**,** S_th**,** ‘MinPeakDistance’**,** min_peak_distance**);**




## 3. Results

Section 3 summarizes the results obtained by the proposed segmentation algorithm for extracting the systolic intervals. In detail, EMAT, PEP, LVET, and LVST were measured and compared with typical physiological ranges based on segmented signals. Also, normalized systolic times and the PEP/LVET ratio were computed, providing further insights into the cardiac function. Also, the validation of the proposed algorithm on signals of an open-source dataset (BSSLAB Localized ECG Dataset) is reported. Furthermore, the following sections detail the methods and findings of this analysis.

### 3.1. Extraction of the Systolic Times from PCG and ECG Processing

**Figure 10:**
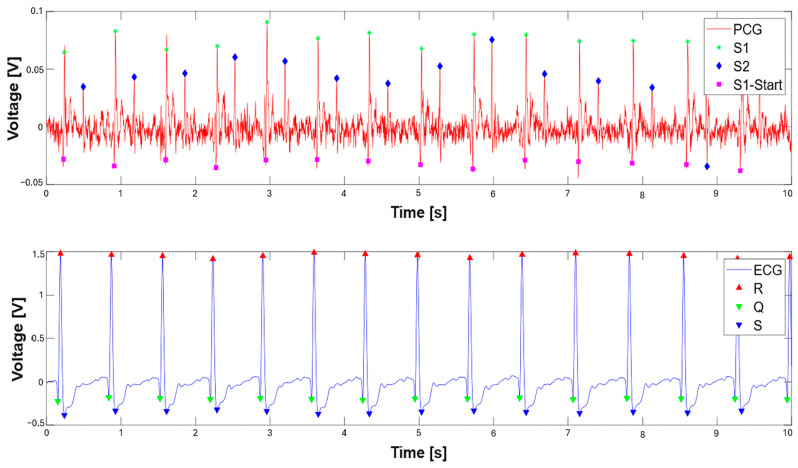
PCG and ECG signals with performed segmentation: S1 (green asterisk), S2 (blue diamonds), and S1 onset sounds (magenta square). Q (green triangle), R (red triangle), and S waves (blue triangle).

Figure 10 presents the concurrently acquired PCG and ECG signals and their corresponding segmentations. Within the PCG signal, the onset of the S1 sound is denoted by a fuchsia square, the peak of the S1 sound by a green asterisk, and the peak of the S2 sound by a blue diamond. In the ECG signal, the Q, R, and S wave peaks are indicated by a green downward-pointing triangle, a red upward-pointing triangle, and a blue downward-pointing triangle, respectively.

**Figure 11:**
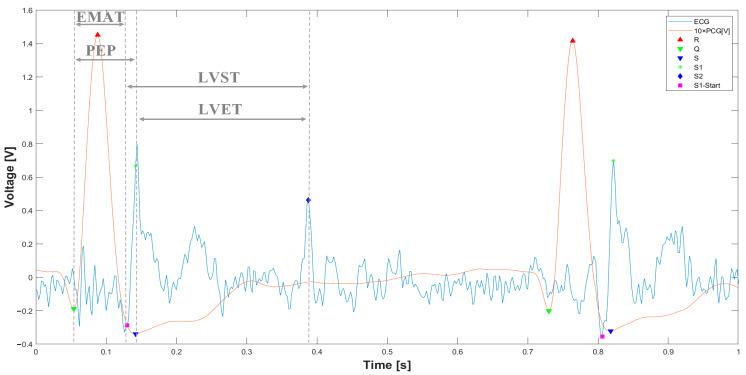
Overlaid ECG and PCG signals with determined systolic times (EMAT, PEP, LVET, and LVST).

After segmenting the PCG and ECG signals, the systolic times are determined, which are highlighted in Figure 11. In detail, the EMAT is reported, i.e., the time between the Q peak and the beginning of the S1 signal, the PEP, which indicates the time between the Q peak and the S1 peak of the PCG, then passing through the LVET, determined as the time between the beginning of the S1 sound and the peak of S2, and, finally, the LVST, which indicates the time elapsed from the peak of the S1 and S2 sounds.

**Figure 12:**
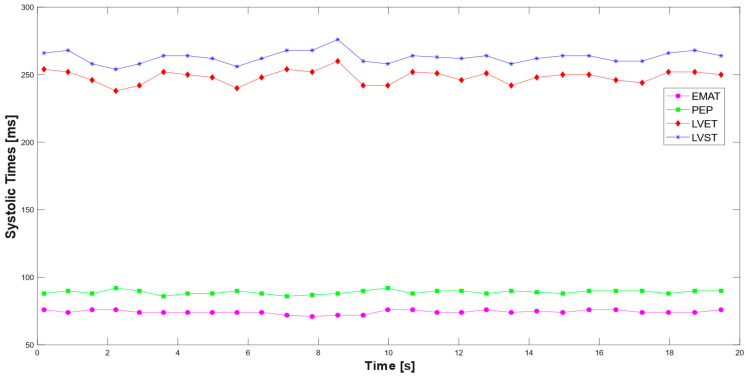
Time trend of systolic times extracted from the analysis of the two signals.

Based on the segmentation performed, the systolic time intervals are measured over a 20 s observation period, during which 28 cardiac cycles are recorded. Figure 12 illustrates the temporal variation of the systolic times across the observed interval.

The mean values of systolic times measured over the observation interval are reported in Table 5. As evidenced, the values determined by the analysis of the PCG and ECG signals agree with the typical ranges of the corresponding parameters. The developed script for ECG and PCG segmentation and the extraction of systolic intervals is available in the Supplementary Material (File S1).
412  % Calculate systolic times 413  EMAT **= (**S1_start**-**TQ2)*1000**;** 414  PEP **= (**S1_time_**-**TQ2)*1000**;** 415  LVET **= (**S2_time_**-**S1_time_)*1000**;** 416  LVST **= (**S2_time_**’-**S1_start)*1000**;** 417  S1R **= (**S1_start**-**TR2)*1000**;** 

**Figure 13:**
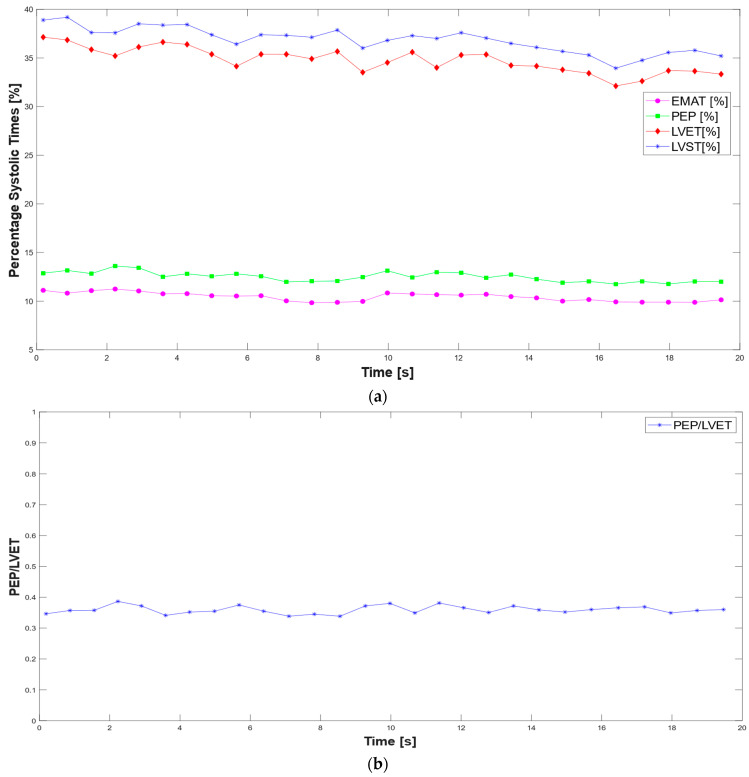
Time trend of normalized systolic times (**a**) and PEP/LVET ratio (**b**) extracted from PCG and ECG analysis.

Finally, the systolic times normalized for the RR interval corresponding to the cardiac cycle are calculated, as well as the ratio between the pre-ejection period (PEP) and the ejection time (LVET) (Figure 13).

(1) $$EMAT\;\left[\%\right]=\frac{EMAT}{RR}$$

(2) $$PEP\;\left[\%\right]=\frac{PEP}{RR}$$

(3) $$LVET\;\left[\%\right]=\frac{LVET}{RR}$$

(4) $$LVST\;\left[\%\right]=\frac{LVST}{RR}$$

### 3.2. Validation of the Proposed Method for Systolic Time Estimation

The proposed algorithm for the estimation of systolic time intervals was applied to an open-source dataset (called BSSLAB Localized ECG Data) consisting of localized ECG signals recorded at PCG auscultation sites, along with respiratory signals [13]. The data, saved in a .mat format with a sampling frequency of 20 kHz, are organized into 12 folders, each corresponding to a different subject. The authors thoroughly analyzed the dataset in [14]; specifically, two experimental phases were conducted: In Phase I, the simultaneous acquisition of ECG signals—using both gel and embroidered electrodes—and PCG signals—using electronic stethoscopes—was carried out at the primary auscultation sites. A microphone placed under the subject’s nose also recorded a respiratory signal. In phase II, ECG signals were acquired at a single auscultation site using disposable adhesive sensors with three different inter-electrode distances (5, 10, and 15 cm). In both phases, the duration of each recording was 3 min. The systolic time intervals obtained with the proposed algorithm were compared with the ECG–PCG delays reported in the reference article [14]. In particular, attention was focused on the time interval between the Q peak and the onset of the S1 sound and between the R peak and the onset of S1.

**Figure 14:**
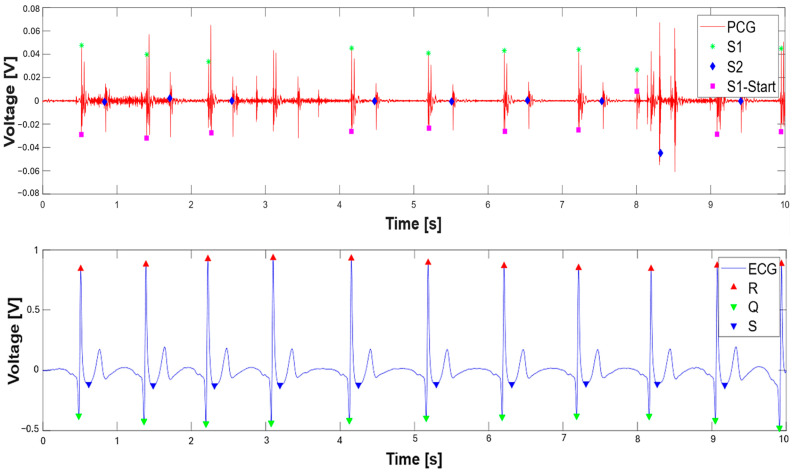
The 10 s segment extracted from one of the signals in the dataset, with PCG and ECG recorded at point A. S1 (green asterisk), S2 (blue diamonds), and S1 onset sounds (magenta square), Q (green triangle), R (red triangle), and S waves (blue triangle).

The same segmentation algorithm previously described was applied to the signals extracted from the dataset, adapted to 10 s windows. The algorithm uses adaptive detection thresholds that vary dynamically based on the characteristics of the signal. Figure 14 shows an example of the segmentation applied to 10 s of ECG and PCG signals recorded at site A. The S1 and S2 sounds were identified, as well as the onset of the S1 sounds, from which two intervals of interest were calculated: the S1–R interval, i.e., the time distance between the onset of S1 and the R peak, and the S1–Q interval, i.e., the distance between the onset of S1 and the Q point. These intervals were analyzed to compare the results obtained with those reported in the reference article [14].

**Figure 15:**
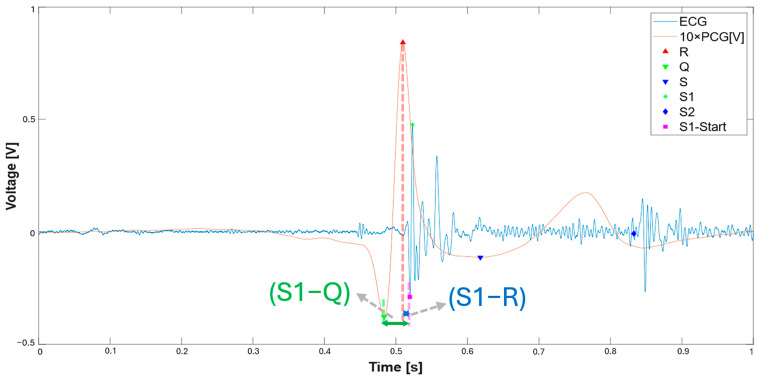
The 10 s ECG and PCG signal recorded at point A, with highlighted the S1–R and S–Q intervals; the first one is the time interval between the onset of the S1 sound of PCG (magenta square) and the R wave of ECG (red triangle), whereas the latter is the time interval between the onset of the S1 sound of PCG (magenta square) and the Q wave of ECG (inverted green triangle).

In particular, Figure 15 highlights the length of the S1–R and S1–Q intervals, as illustrated in the original study, in which the interval between the onset of S1 and the Q point corresponds to EMAT.

**Figure 16:**
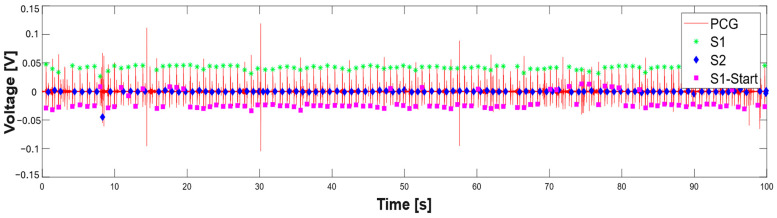

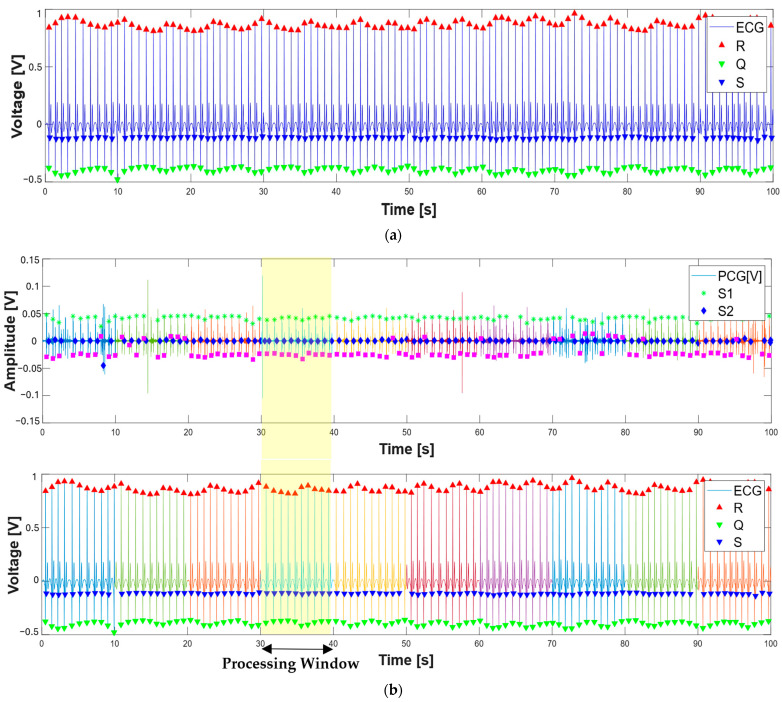
Segmented PCG and ECG signals lasting 100 s: segmentation results without window indication (**a**) and highlighting consecutive 10 s processing windows in different colors (**b**). S1 (green asterisk), S2 (blue diamonds), and S1 onset sounds (magenta square), and Q (green triangle), R (red triangle), and S waves (blue triangle).

Subsequently, the segmentation procedure used for the individual 10 s windows was extended to ECG and PCG signals lasting 100 s extracted from the dataset, thus allowing for a more accurate evaluation of the temporal characteristics of the PCG and ECG signals. Figure 16a presents the segmented PCG and ECG signals using the proposed algorithm, where the characteristic points (such as Q, R, and S waves and the S1 and S2 sound onset) are clearly marked. In addition, Figure 16b highlights consecutive 10 s windows using different colors, illustrating that the code processes the signals in fixed-length segments. This processing approach improves the segmentation accuracy by limiting the analysis to smaller, more stable time intervals, thereby reducing the impact of signal variability over longer recordings.

## 4. Discussion

As previously described, the LVET is defined as the time interval between the opening and closing of the aortic valve and corresponds to the systolic phase during which the left ventricle ejects blood through the aorta. The LVET has been used for decades to assess left ventricular function and contractility. However, most recently, the LVET has been used as a measure of the therapeutic action of new drugs in patients with heart failure with a reduced ejection fraction (HFrEF), since the LVET is diminished in these patients [35]. Some studies have shown that in HFrEF patients, the LVET decreases while the PEP increases, compared to healthy individuals [36]. The increase in the PEP can be attributed to a decreased left ventricular pressure rate (dP/dt) during isovolumetric contraction. Furthermore, in patients with HFrEF, myocardial fiber shortening is reduced, leading to a reduction in the LVET [37].

Thus, the reduction in the contractile capacity of the left ventricle will lead to an increase in the PEP/LVET ratio. To expand on the clinical implications of these systolic time intervals, another study highlighted the clinical relevance of the LVET and EMAT% in acute myocardial infarction (AMI) patients. This study shows that EMAT% > 12.1% correlates with a lower left ventricular ejection fraction (LVEF), prolonged EMAT and PEP, and reduced LVET. These changes were associated with an increased LVEDV and LVESV, indicating a worsening ventricular function. Additionally, EMAT% > 12.1% was linked to a higher incidence of MACE (Major Adverse Cardiovascular Events), particularly in post-STEMI (ST-elevated myocardial infarction) patients [34].

Additional studies further support the role of the EMAT and %EMAT in evaluating the left ventricular function. The EMAT, defined as the time from the ECG Q peak to the first peak of S1, and %EMAT, calculated as the EMAT divided by the RR interval, have been investigated as diagnostic markers. Research has demonstrated that an EMAT ≥ 104 ms predicts an LVEF < 50% with high sensitivity and specificity [38]. Complementing this, another study reported that a %EMAT > 13.8% was significantly associated with a reduced ejection fraction and a higher risk of in-hospital major adverse cardiac events (MACE) in heart failure patients, underscoring the prognostic value of this parameter [39]. Further reinforcing the diagnostic utility of systolic time intervals, another study demonstrated that an EMAT/LVST ratio ≥ 0.40 is indicative of left ventricular dysfunction, particularly in patients with an elevated LVEDP and reduced LVEF. Their study supports the use of combined phonoelectrocardiographic and biochemical markers to enhance the diagnostic accuracy [40]. Additionally, a paper highlighted significant correlations between the PEP, LVET, and ejection fraction (EF) in patients with ischemic heart disease and mitral valve disease. Specifically, the PEP was inversely correlated with the EF (r = −0.69), while the LVET showed a positive correlation (r = 0.67). Moreover, the PEP/LVET ratio was even more strongly correlated with both the EF (r = −0.77) and stroke index (r = −0.72), with subsequent findings in mitral valve disease showing a correlation as high as r = −0.87. These results emphasize the broader clinical value of systolic time interval ratios across different cardiac pathologies [41]. Combining multiple indicators, such as the EMAT, EMAT/LVST, and S3 scores, improves the diagnostic accuracy over single measurements. Furthermore, the night-time EMAT is a significant predictor of post-discharge events, suggesting its potential role in long-term risk assessment [42]. The mechanism behind a shortened LVET in the HFrEF is complex. Studies indicate that while the myocardial fiber shortening velocity is reduced in HFrEF, the overall extent of fiber shortening is also diminished. This reduction in the shortening extent outweighs the expected prolongation of the LVET, leading to a net decrease in the LVET. A prolonged PEP due to an impaired LV pressure rise (dP/dt) further delays the ejection onset, reducing the LVET. As this last is directly related to the stroke volume, a shortened LVET in HFrEF correlates with a decreased stroke volume and cardiac output, reinforcing its role as a critical parameter in assessing systolic dysfunction [43]. Technological innovations have increasingly focused on simplifying the assessment of systolic function; to this end, a real-time automated system was proposed to evaluate the systolic cardiac performance using ECG and PCG signals. Integrating a modified digital stethoscope and a custom MATLAB application, their system successfully measured the PEP, LVET, and PEP/LVET ratio. Testing on healthy individuals showed high agreement with echocardiographic results, indicating potential use for the long-term monitoring of congestive heart failure patients. The system’s ability to automate and simplify systolic function assessments highlights its potential for non-invasive and continuous cardiac monitoring [44]. Together, these findings highlight the potential of the EMAT% in improving risk stratification, particularly for AMI and HFrEF patients, while reinforcing the LVET as a crucial marker of systolic dysfunction. Integrating PCG and ECG in non-invasive monitoring may improve the cardiovascular disease management.

To consolidate the clinical significance of systolic time intervals, Table 6 reports a structured correlation between selected cardiac pathologies and corresponding alterations in the EMAT, PEP, LVET, and LVST values; where applicable, diagnostic thresholds derived from the literature are included to support the interpretation. This synthesis provides a practical reference for identifying systolic dysfunction and stratifying cardiovascular risk based on non-invasive temporal biomarkers extracted from synchronous ECG and PCG signals.

The proposed system is well-suited for real-time implementation due to its computationally efficient segmentation algorithm and low-complexity hardware architecture, which enables rapid processing and minimal latency. Its modular design facilitates integration into portable and wearable platforms, making it a viable candidate for continuous ambulatory monitoring. The segmentation algorithm, based on the signal morphology and temporal features, requires minimal computational resources and can be deployed on embedded systems or low-power microcontrollers without a significant compromise in the accuracy or speed. However, several challenges must be addressed to ensure a robust real-time performance across diverse environments. The system’s accuracy may degrade in the presence of substantial motion artifacts, irregular heart rhythms (e.g., arrhythmias), or a poor signal quality—common issues during patient movement, speech, or in noisy clinical or home settings. These factors can distort the temporal alignment between ECG and PCG signals, reduce the signal-to-noise ratio, and lead to the erroneous segmentation of cardiac events. To mitigate these limitations, future development could incorporate real-time signal quality assessment modules to detect and flag unreliable segments dynamically. Advanced denoising techniques, such as wavelet-based filtering, empirical mode decomposition, or adaptive filtering algorithms (e.g., LMS, RLS), may be implemented to suppress artifacts while preserving relevant physiological features.

Additionally, integrating accelerometer or gyroscope data could facilitate motion artifact correction through sensor fusion approaches. Machine learning or deep learning models trained on diverse datasets could also enhance the segmentation accuracy, especially in complex scenarios involving pathological conditions or noisy environments. Moreover, a feedback-driven calibration phase could be added to adjust the algorithm parameters based on individual-specific cardiac dynamics, improving the personalization and long-term monitoring performance. In summary, while the system demonstrates strong potential for real-time and portable cardiac monitoring, addressing motion resilience and adaptability to diverse physiological and environmental conditions will be critical for its widespread deployment. Continued research into robust signal processing and intelligent artifact management will further enhance the clinical utility and reliability of the system in real-world settings. Compared to conventional clinical methods—such as echocardiography or catheter-based measurements—the proposed system offers several advantages: it is non-invasive, cost-effective, and portable, making it suitable for continuous or remote monitoring outside clinical settings. Additionally, its ability to provide real-time beat-to-beat systolic timing analysis can enhance the early detection and longitudinal tracking of cardiac dysfunction, particularly in resource-limited or home-based care environments.

In conclusion, this study demonstrates the feasibility of synchronized ECG and PCG signals for the real-time assessment of systolic cardiac function. The developed method offers a promising alternative to traditional diagnostic tools, with potential applications in clinical and wearable health monitoring contexts.

### Analysis of Numerical Results for Validation of the Proposed Algorithm for Systolic Time Measurement

In this section, the comparison of systolic intervals (Q–S1 and R–S1) obtained by the proposed algorithm in [14] are reported to quantify its performance.

**Figure 17:**
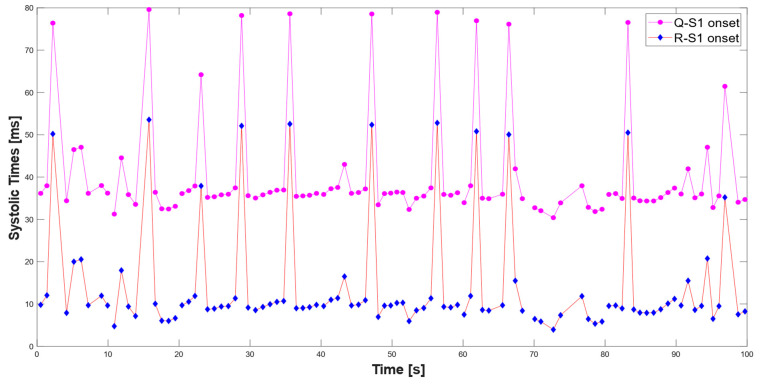
Time trends of Q–S1 and R–S1 intervals over a 100 s observation period.

Figure 17 illustrates the time evolution of the systolic intervals, specifically the Q–S1 and R–S1 times, over a 100 s observation period. Mean values were computed using the developed MATLAB code, and strong agreement was demonstrated with those reported in the reference study [14]. In particular, the extracted mean value of the Q–S1 interval was 40.45 ms (standard deviation: 11.52 ms), closely matching the reported value (≈50 ms), and similarly, the mean value of the R–S1 interval was 14.09 ms (standard deviations: 4.63 ms), both consistent with the reference values (≈15 ms) for the corresponding PCG auscultation site and ECG acquisition setup. The interquartile ranges were 8.9 ms for the Q–S1 times and 3.4 ms for the R–S1 times, whereas the median values were 38.3 ms and 13.7 ms, respectively. The resulting mean S1–Q interval aligns with the range reported for healthy subjects using gold-standard methods (manual annotation, 35–80 ms) in the reference study [45].

The relatively low R–S1 and Q–S1 intervals variance suggest that these metrics may serve as a more stable surrogate marker for electromechanical activation timing, especially in rhythmically regular segments. These findings support the robustness of the implemented detection algorithms and the physiological plausibility of the measurements. Physiologically, both intervals reflect the electromechanical delay—the time from electrical depolarization to mechanical contraction—and their values are influenced by an autonomic tone, preload conditions, and signal processing parameters. Occasional outliers in the Q–S1 and R–S1 intervals appear to be associated with transient fluctuations in the ECG and PCG signal morphology and artifacts, which may compromise the detection accuracy. However, their presence can be reduced by further refining the segmentation algorithm by integrating mechanisms to control the acceptability of the detected PCG and ECG peaks.

**Figure 18:**
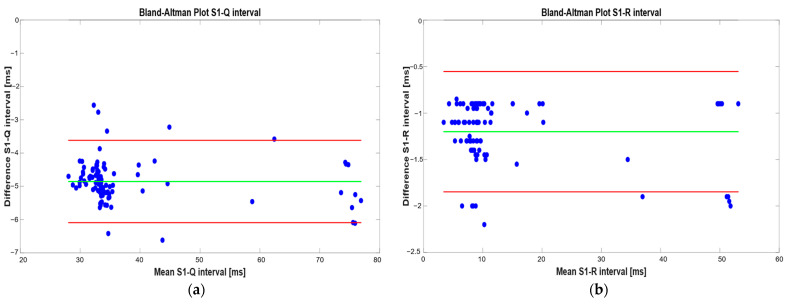
Bland–Altman plots related to (S1–Q) (**a**) and (S1–R) (**b**) intervals by means of developed algorithm and using dataset reported in [14]; the blue points represent the individual data measurements (difference vs. mean), the green line indicates the mean difference, and the red lines denote the limits of agreement, showing the range within which 95% of the differences are expected to fall.

The proposed algorithm was validated using signals acquired with hospital equipment, including a bench-top electrocardiograph and a phonocardiograph [13]; the data, made available through an open-source dataset, were collected within the study described in [14], carried out by an interdisciplinary team of engineers and physicians affiliated with King’s College London. The reference results from this study were clinically validated by King’s College Hospital medical staff. Collaboration with the authors for the reference work allowed for a detailed understanding of the acquisition and validation procedures. The close agreement between the systolic intervals (S1–Q and S1–R) obtained with the proposed method and those reported in [14] confirms the reliability and accuracy of the developed algorithm. Furthermore, we reproduced the segmentation algorithm reported in [14] to derive the error metrics. Figure 18 shows the Bland–Altman plot of S1–Q and S1–R intervals obtained with the two methods.

The metrics describing the performance of the proposed algorithm with respect to the reference method are summarized in Table 7.

The Bland–Altman analysis revealed a mean difference of −4.85 ms for the S1–Q interval, with limits of agreement ranging from −6.09 ms to −3.42 ms. The mean difference for the S1–R interval was −1.20 ms, with limits of agreement between −1.85 ms and −0.55 ms. Furthermore, the S1–R measurement outperforms S1–Q in both the average (1.20 ms vs. 4.85 ms) and worst-case error (2.20 ms vs. 6.64 ms), reinforcing its higher reliability. The worse performance in the S1–Q measurement with respect to the S1–R one is probably ascribable to the non-overlapping identification of the Q peaks between the two methods. Despite a lower absolute error, in some cases, the S1–R interval might become inaccurate, perhaps when the R-wave amplitude or timing varies substantially. The relative error metrics further support these findings: the S1–Q interval shows a lower mean relative error (8.65%) compared to the higher relative deviation observed in S1–R (12.05%), likely due to the typically shorter duration of S1–R making it more sensitive to small absolute shifts. Similarly, the higher maximum relative error in S1–R (18.60%) suggests that, although generally more accurate, this measurement can be more affected by atypical signal morphologies. The results confirm the agreement between the two measurement methods, validating the reliability of our segmentation algorithm.

## 5. Conclusions

This study presented a system for the simultaneous acquisition and analysis of ECG and PCG signals, enabling a more integrated assessment of cardiac function by combining the heart’s electrical and mechanical components. The system includes an AD8232 front-end for ECG recording, a digital stethoscope for PCG acquisition, and an Arduino Nano BLE board for signal acquisition and export. An adaptive segmentation algorithm was developed and applied to synchronously acquired ECG and PCG signals, enabling the automatic detection of relevant features (Q, R, and S peaks and S1 and S2 sounds) and the extraction of systolic time intervals. The proposed method returned average values of EMAT = 74.35 ms, PEP = 89.00 ms, LVET = 244.39 ms, and LVST = 258.60 ms, which fall within physiological ranges, supporting the robustness of the approach. The clinical relevance of these intervals has been established in the previous literature; for instance, the EMAT% can be used for risk stratification in acute myocardial infarction, while the LVET is a key marker of systolic dysfunction.

To assess the generalizability of the approach, the algorithm was also tested on the BSSLAB Localized ECG Dataset, yielding mean values of 40.45 ms for Q–S1 and 14.09 ms for R–S1 delays, in agreement with the reference values (mean difference −4.85 ms and −1.2 ms, respectively) reported in [14]. Future work will focus on integrating real-time processing capabilities, expanding testing across a broader population, and refining the hardware design to improve the portability and usability. While the system shows potential for educational, research, and clinical applications, particularly in non-invasive cardiovascular monitoring, further validation will be required to ensure its reliability in real-world diagnostic settings.

## Figures and Tables

**Table 1:** Summary table of previously discussed PCG sensors.

Reference	Construction Technology	Materials Used	Application Area	Parameters/Extracted Signals	Sensitivity
J. Cui et al. [15]	Cantilever piezo-resistors	Si, SiO_2_, low B ion, high B ion, Au	Thorax	PCG signal voltage (V)	189.5 dB @ 500 Hz
T. Wang. et al. [16]	Capacitive electret microphone	Layer electret	Thorax	PCG signal array	N. A.
S. Lee et al. [17]	MEMS microphone and PPG sensor	Silicon	Finger and chest	PPG and PCG signals simultaneously	N. A.

N. A.: not available.

**Table 2:** Summary table of the previously discussed algorithms.

Reference	Approach/Model	Output Classes	Input Features	Accuracy
C. Yin et al. [18]	ResNet and attention module	Resting/post-exercise	Durations of segments S1, S2, S3, S4	98%
S. Behera et al. [19]	DT, RF, SVM, KNN	Severe, Mild AS, Mild, Moderate MS, Healthy	DWT, WPT, and PWPT features	99.2%
Z. Tariq et al. [20]	FDC-FS	Multi-classification CVD (normal, AS, MR, MS, MVP)	Multi-features images	97%
Y. Huang et al. [21]	Clustering, feature fusion, RF	Normal/CAD	MFCC and PCG signal segments	96.05%
H. Li et al. [22]	CNN-1D CNN-2D	Normal/CAD	Signals, spectral images, and time–frequency images of ECG and PCG	96.51%
S. Ma et al. [23]	CNN-1D	Normal/VHD	Features generated from convolutional layers	99.4%
H. K. Alkahtani [24]	CNN-1D	Normal/VHD	Features generated from convolutional layers	98.56%

**Table 3:** Summary table of systems for acquiring and processing PCG and ECG signals previously discussed.

Reference	PCG Acquisition Channels	Type of PCG Sensor	Application Area	Additional Signals	Algorithm	Type of Measurement/Classification
Y. Rong et al. [25]	12 in total (6 heart-sensor microphones and 6 background noise microphones)	N. A.	Chest	ECG	CNN-based CVD classifier using both the PCG and ECG signals	Classification Normal/CVD
N. Giordano et al. [26]	48	Electret microphone and MIMU	Chest	ECG	-	Classification Normal/CVD
P. Jyothi et al. [28]	1	N. A.	N. A.	ECG	GKVDLNN	Classification arrhythmias, ischemic, prolapsed mitral valve, normal, valvular heart diseases
S. Gnanapira-kasam et al. [29]	1–4	Littmann^®^ Electronic Stethoscope Model 3200	N. A.	ECG	CNN-LSTM	Abnormal/Normal ECG, PCG, or both ECG and PCG
M. Klum et al. [30]	1	Digital stethoscope	Chest	ECG	TDNN	Regression Systolic times

N. A.: not available.

**Table 4:** Technical specifications of the HMYL HM-9250 electronic stethoscope.

Pickup range	0–20,000 Hz
Sensitivity	−52 dB
Noise Level	Less than −45 dB
Magnification	4×
Bias voltage	DC 2.6–3 V
Signal	Analog
Signal range	0–200 mV
Sampling rate	44.1 KHz
Interface standard	National Standard or American Standard

**Table 5:** Mean values of the determined systolic times and corresponding typical ranges.

Systolic Times	Measured Systolic Times	Typical Range
EMAT [ms]	74.35	50–120 [31]
PEP [ms]	89.00	50–120 [32]
LVET [ms]	244.39	150–350 [33]
LVST [ms]	258.60	150–350 [34]

**Table 6:** Correlation between cardiac pathologies and systolic time parameters.

Pathology	EMAT [ms]	PEP [ms]	LVET [ms]	LVST [ms]	Clinical Insight/Thresholds
Acute myocardial infarction (AMI) [29]	Increased (EMAT% > 12.1%)	Increased (%PEP from 16.23 to 17.29)	Reduced (%LVET from 30.63 to 29.85)	Reduced (%LVST from 38.07 to 37.38	Associated with lower LVEF, increased LVEDV/LVESV, and higher MACE risk.
Depressed left ventricular ejection fraction (dLVEF) [38]	Increased (EMAT ≥ 104 ms)	-	-	-	EMAT ≥ 104 ms predicts LVEF < 50%.
Heart failure with reduced EF (HFrEF) [39]	Increased (% EMAT > 13.8%)	-	-	-	% EMAT > 13.8% predicts LVEF < 50%.
Left ventricular dysfunction [40]	≥15	-	-	-	EMAT/LVST ≥ 0.40 is an indicator of impaired systolic performance.
Ischemic heart disease [41]	-	Increased (r = −0.69 with EF)	Reduced (r = 0.67 with EF)	-	PEP/LVET is correlated with the Ejection Fraction (EF) and Stroke index with correlation r = −0.77 and −0.72.
Mitral valve disease [41]	-	Increased (r = −0.69 with EF)	Reduced (r = 0.67 with EF)	-	PEP/LVET is correlated with the Ejection Fraction (EF) with a correlation r = −0.87.

**Table 7:** Metrics of S1–Q and S1–R measurements of proposed methods with respect to the reference.

Metric	S1–Q Interval	S1–R Interval
Mean difference [ms]	−4.85	−1.20
LoAs [ms]	−3.42/−6.09	−0.55/1.85
Mean absolute error [ms]	4.85	1.20
Maximum absolute error [ms]	6.62	2.20
Mean relative error [%]	8.65	12.05
Maximum relative error [%]	10.39	18.60

## Data Availability

The original contributions and data presented in this study are included in the article. Further inquiries and requests can be directed to the corresponding author.

## Supplementary Materials

The following supporting information can be downloaded at: https://www.mdpi.com/article/10.3390/s25134220/s1, File S1: Script_Matlab.

## Abbreviations

The following abbreviations are used in this manuscript:

ECG	Electrocardiography
PCG	Phonocardiography
EMAT	Electromechanical Activation Time
PEP	Pre-Ejection Period
LVET	Left Ventricular Ejection Time
LVST	Left Ventricular Systolic Time
S1	First Heart Sound
S2	Second Heart Sound
S3	Third Heart Sound
S4	Fourth Heart Sound
MEMS	Micro-Electromechanical Systems
ECM	Electret Condenser Microphones
MCU	Microcontroller Unit
FPGA	Field-Programmable Gate Array
FIR	Finite Impulse Response
SNR	Signal-to-Noise Ratio
PPG	Photoplethysmogram
EICF	Exercised-Induced Cardiac Fatigue
DL	Deep Learning
DWT	Discrete Wavelet Transform
LR-HSMM	Logistic Regression Hidden Semi-Markov Model
FDC-FS	Feature-Based Fusion Model
MFCC	Mel-Frequency Cepstrum Coefficient
CNNs	Convolutional Neural Networks
CAD	Coronary Artery Disease
ResNet	Residual Neural Network
AS	Aortic Stenosis
MR	Mitral Regurgitation
MS	Mitral Stenosis
MVP	Mitral Valve Prolapse
RF	Random Forest
CVD	Cardiovascular Disease
CHF	Chronic Heart Failure
CTIs	Cardiac Time Intervals
GKVDLNN	Gaussian Kaiming Variance-based Deep Learning Neural Network
IEMD	Improved Empirical Mode Decomposition
PEP	Pre-Ejection Period
TDNN	Time Delay Neural Network
CMRR	Common Mode Rejection Ratio
LED	Light-Emitting Diode
SoC	System on Chip
IoT	Internet of Things
BLE	Bluetooth Low Energy
IVS	Interventricular Septum
HFrEF	Heart Failure with Reduced Ejection Fraction
AMI	Acute Myocardial Infarction
LVEF	Left Ventricular Ejection Fraction
MACE	Major Adverse Cardiovascular Events
STEMI	ST-elevated myocardial infarction
LVEDP	Left Ventricular End-Diastolic Pressure
